# Predictors, Healthcare Utilization and Costs Related to Short-Term Stays in Patients with COPD: A Registry-Based Analysis in Norway

**DOI:** 10.2147/CLEP.S521958

**Published:** 2025-08-26

**Authors:** Tron Anders Moger, Jon Helgheim Holte, Olav Amundsen, Silje Bjørnsen Haavaag, Øystein Døhl, Line Kildal Bragstad, Ragnhild Hellesø, Trond Tjerbo, Nina Køpke Vøllestad

**Affiliations:** 1 Department of Health Management and Health Economics, Institute of Health and Society, University of Oslo, Oslo, Norway; 2 Department of Public Health Science and Interdisciplinary Science, Institute of Health and Society, University of Oslo, Oslo, Norway; 3 Department of Finance, Municipality of Trondheim, Trondheim, Norway; 4 Department of Neuromedicine and Movement Science, Faculty of Medicine, Norwegian University of Science and Technology, Trondheim, Norway; 5 Department of Rehabilitation Science and Health Technology, Oslo Metropolitan University, Oslo, Norway

**Keywords:** home-based services, COPD, registry data, healthcare utilization, long-term care, healthcare costs

## Abstract

**Background:**

Chronic obstructive pulmonary disease (COPD) incurs significant healthcare costs, often accompanied by multimorbidity. Advanced patients may need short-term stays for rehabilitation, treatment, or respite to maintain home living.

**Aim:**

To identify predictors for a first short-term stay and study the healthcare utilization and costs compared with similar patients without a short-term stay.

**Patients and Methods:**

Data on COPD patients in the cities Oslo and Trondheim 2010–2019 and including information on specialist, primary and long-term care, diagnoses, sociodemographics and -economics were collected from national and municipal registries, resulting in a sample of 24,613 patients. Using discrete time survival models, we identified predictors for a short-term stay. We described the costs before and after admission, and the duration of living at home, compared to non-recipients matched on age, comorbidities and healthcare use.

**Results:**

Depression, anxiety, mental disorders, alcoholism, prior hospitalization and reception of home care were associated with higher odds of short-term stays. One to two GP visits for respiratory diseases, being in the top quartile for GP visits for non-respiratory diseases, visits to specialists, and physiotherapist visits for non-respiratory issues were significantly associated with lower odds of short-term institutional stay. Patients admitted to short-term stays incurred markedly higher costs both in the year before admission and during subsequent years compared to matched non-recipients, primarily due to increased use of inpatient and home care services.

**Conclusion:**

Prior receipt of home care, unlike standard outpatient services, was linked to a higher likelihood of short-term stays. This suggests that some outpatient services may delay the need for such stays, or that patients already in municipal services are more readily admitted. Additionally, patients with psychosocial issues may have greater care needs, indicating that resource allocation aligns with these needs. The findings suggest that by the time short-term stays are required, health deterioration has already become considerable.

## Introduction

Chronic obstructive pulmonary disease (COPD) is a common heterogenous and progressive disease characterised by persistent respiratory symptoms and airflow limitation to the lungs, which over time may result in structural changes in the airways.^1^ COPD patients are typically elderly and commonly suffer from several comorbidities, and the condition is associated with major healthcare costs worldwide.^2–5^ Estimates for the total macroeconomic burden of COPD are as high as more than $4 trillion during 2020–2050.^2^ In addition, it is one of the leading causes of death, responsible for over three million fatalities and impacting more than 200 million individuals in 2019.^6^,^7^

Direct healthcare costs of COPD have been described at the macro-level.^3–5^ As highlighted by Faes et al,^8^ the composition of costs at the patient level, including home- and institution-based long-term care, has not been extensively studied in the literature. An earlier study in the US indicates that the costs of long-term care among the oldest COPD population are substantial,^9^ with significantly higher costs for COPD patients compared to demographically similar non-COPD patients.^10^ However, in many countries accessing individual-level long-term care data is challenging due to limited availability, which could explain the lack of studies.^11^,^12^ Furthermore, we are not aware of any studies that examine health service use before or after a short-term institutional stay. This applies to the COPD population, but also seems to be the case more generally.^13^

In Norway, long-term care—both home-based and institutional—is provided by the municipalities, either free of charge or financed through limited income-dependent co-payments. All residents can apply for long-term care services without the need for a referral from health professionals. However, the municipalities have the authority to decide the need, and the amount and type of care. Home care covers health and medical services, as well as general assistance and equipment to be able to live at home as the functional level declines. Temporary institutional care, commonly known as short-term stays, provides treatment as well as monitoring of the patient. In Norway, the Health and Care Services Act mandates access to short-term stays in all municipalities. Short-term stays are provided when more complex, but temporary, care needs arise and is also frequently used as a step in the transition of the patient from hospital to home. The motivation for this paper arises from earlier findings indicating that many new recipients of municipal health services in Norway begin their care journey at a high level,^14^,^15^ and the presumption voiced by municipal healthcare providers and managers that health declines particularly rapidly after a first short-term stay. The decline likely reflects the patient’s already poor health status and trajectory towards further health deterioration and increased need for healthcare services.

Healthcare data in Norway can be linked at the individual level, encompassing primary care, specialized care (both inpatient and outpatient), and long-term care. Utilizing these extensive data, it is of interest to identify healthcare use and patient characteristics that predict admission to a first short-term stay. Specifically, we will focus on variables related to contact with different outpatient services and factors associated with low socio-economic status, such as mental disorders, alcoholism, and disability pensions—as social vulnerability is more prevalent among COPD patients than in the general population.^1^ The analysis may not only help identify whether there is social inequality in access to short-term stays within the COPD population but also highlight the types of services associated with a reduced likelihood of a first short-term stay. If a first short-term stay is indeed linked to a rapid increase in healthcare resource use or health deterioration, understanding these factors could inform the discussion on how to potentially delay the progression to permanent nursing home admission or death.

Thus, the aim of the paper is first to identify factors associated with both higher and lower likelihood of a first short-term stay. Second, we aim to compare the healthcare use, costs, and time living at home for patients admitted to a first short-term stay versus patients not admitted. This paper builds on earlier work that described the in- and outpatient service use before and after becoming a recipient of any home care from the municipality.^15^

## Materials and Methods

### Sample Selection

This study was conducted as part of the Innovations in use Of REGistry data project (INOREG), administered by the Department of Health and Society, University of Oslo. All patients residing in Oslo or Trondheim at any time between 2010 and 2019 with at least one registered COPD diagnosis in primary or secondary care were included. Diagnoses were identified using the International Classification of Diseases, 10th Revision (ICD-10): J43, J44, and the International Classification of Primary Care (ICPC-2): R95, sourced from the Norwegian Patient Registry (NPR) and the Control and Payment of Reimbursement to Health Service Providers (KUHR). Oslo and Trondheim are, respectively, the largest and third largest cities in Norway, with approximately 720 and 215 thousand inhabitants. The first registered COPD diagnosis in the sample can occur at any point during a patient’s time living with the disease. For the majority of patients, the date of the first diagnosis recorded in the sample may be several years after their actual first COPD diagnosis (if it occurred prior to the inclusion period). For some patients, it may coincide with their initial recorded COPD diagnosis, making them incident cases in the sample. Therefore, the data represent a broad snapshot of the general diagnosed COPD population in the two municipalities.

Data were linked at the individual level to sociodemographic and -economic data from Statistics Norway, time of death from the Cause of Death Registry, and to long-term care data from the municipality electronic patient journal in Oslo and Trondheim. The database includes information on health care use and diagnoses from 2008. Thus, we required patients to have no registered short-term stays during 2008–9 to be included in the sample. The vast majority with a first short-term stay registered any time during 2010–2019 should then be considered incident recipients. The sample was further restricted to individuals above 40 years of age and censored at 95 years of age. This resulted in a sample of 24,613 individuals. More information about the project is available elsewhere.^16^

### Dependent Variables

For the first aim, the dependent variable was first short-term stay in institution from Jan 2010 through Dec 2019. For the second aim, we describe direct healthcare use and costs in primary care (including GP visits, physiotherapy, home-based care, and short-term institutional care) and specialist care (both in- and outpatient) before and after the first short-term stay. Additionally, we examine the time to permanent placement in a nursing home or death. Both permanent nursing home admission and death are absorbing states. Even though high costs are incurred following admission to a nursing home, it is no longer realistic to improve the patient’s health to the extent that they can be transferred back home.

### Independent Variables

The list of independent variables and data sources is given in Table 1. In order to distinguish between care related to respiratory diagnoses and other types of care, all in- and outpatient contacts were categorised according to having a respiratory main diagnosis (ICD-10: J, ICPC-2: R) or non-respiratory diagnoses. GP, physiotherapist, contract specialist and emergency room services provided by the municipality require a primary diagnosis for reimbursement, which results in most contacts with these services having only one diagnosis. We have data on contacts with physiotherapists contracted with the municipality and independent specialists contracted with a regional health authority. The number of fully private specialists in Norway is limited and not available in the records. For in- and outpatient hospital care, as well as rehabilitation services provided by hospitals (both public and private under contract with a regional health authority), we defined respiratory contacts as having ICD-10 code J as one of the first two diagnoses. Hospital admissions were considered as one contact if the discharge and admission dates were less than a day apart.

**Table 1:** List of Independent Variables Used in the Analyses

Variable	Definition	Source
**Frequency of health service contacts per year**		
Contacts with GP	Number of contacts and categories in four groups	KUHR
Emergency room contacts	Number of contacts with municipal emergency room and indicator of use yes/no	KUHR
Contact with contract specialist	Number of contacts and indicator of use yes/no	KUHR
Contact with physiotherapist	Number of contacts with general physiotherapist, manual physiotherapist, occupational therapist, and indicator of use yes/no	KUHR
Outpatient contact with hospital	Number of contacts and indicator of use yes/no	NPR
Rehabilitation in hospital or private clinics on public contract	Number of stays and indicator of use yes/no	NPR
Inpatient hospital admissions	Number of admissions and indicator of use yes/no	NPR
**Home care service indicators**		
Safety alarm	Yearly indicator of receiving service	MEPJ
Any assistance at home	Yearly indicator of receiving service and number of hours	MEPJ
Assisted living	Yearly indicator of receiving service and number of days	MEPJ
Respite care in/out of institution	Yearly indicator of receiving service and number of days	MEPJ
Home nursing	Yearly indicator of receiving service and number of hours	MEPJ
Short-term rehabilitation/treatment out of institution	Yearly indicator of receiving service and number of days	MEPJ
**Multimorbidity, sociodemographic and –economic variables**		
Comorbidities	Yearly indicators for each of 17 comorbidities, see Table S1 for details	NPR and KUHR
Age	Age in years	SSB
Gender	Indicator of male gender	SSB
Income	Annual gross income per year	SSB
Education when entering sample	Categorized as primary, secondary and university/college	SSB
Marital status when entering sample	Categorized as married/cohabitant (reference), widow/widower, not married/divorced	SSB
History of disability pension	Indicator for current or pre-retirement receipt of a permanent disability pension.	SSB

**Abbreviations**: NPR=Norwegian Patient Registry, KUHR=Control and Payment of Reimbursement to Health Service Providers, RGP=Regular General Practitioner registry, MEPJ=municipality electronic patient journal, SSB=Statistics Norway.

In addition to variables on healthcare use, we defined indicators for the types of long-term care services provided by the municipality. We also included sociodemographic and –economic variables such as gender, income, education, indicator of current or pre-retirement receipt of a permanent disability pension and marital status from Statistics Norway, and a list of comorbidities commonly associated with COPD^17^ identified from ICD-10 and ICPC-2 codes in NPR and KUHR (Supplementary data, Table S1). We constructed indicator variables for the presence of each comorbidity based on its registration in yearly healthcare contacts.

### Statistical Analysis

The data were structured per year of follow-up, starting from the date of the first registered COPD diagnosis. Descriptive statistics are given as mean (standard deviation) for continuous variables and as percentages (number of patients) for categorical variables. Due to varying follow-up time, the descriptives are presented as means (SD) per follow-up year per patient for the continuous variables. For patients with short-term stays during follow-up, we present descriptives both for periods before and after the first short-term stay.

To analyze risk factors for short-term stays while accounting for the varying follow-up times, we applied discrete time logit survival models and presented hazard odds ratios as in a previous paper,^15^ updating independent variables yearly where relevant. For patients who began follow-up in 2010, we adjusted for the independent variables using data that extended into 2009. To capture the effects of aging on health and the risk of institutionalisation, we used patient age as the time scale. Age was included as yearly dummy variables. Censoring occurred at end of follow-up, permanent nursing home placement or death, whichever occurred first. Regarding the independent variables, GP contacts with respiratory diagnoses were categorized into 0, 1–2, 3–4, and 5 or more contacts per year. Contacts with non-respiratory diagnoses were categorized into 0–2, 3–7, 8–14, and 15 or more contacts per year. The former was based on distinguishing person-years without any GP check-up for respiratory conditions, 1–2 routine check-ups, and a higher number of contacts. The latter was divided according to quartiles in the distribution of the number of contacts. We thus assumed that the difference in health status between having one versus two GP contacts in a year is modest. However, having eg, two vs nine contacts in a year likely reflects significantly worse health in the latter group compared to the former. Home-based services, in- and outpatient hospital, specialist and physiotherapy contacts were split into no/at least one contact. The use of these services occurred much less frequently in the sample, hence it should be sufficient to include indicators of whether these services were used during the year. Comorbidities were included as individual indicator variables in the model.

To describe the healthcare use and costs for patients with a first short-term stay during follow-up, we present means (SD) per follow-up year per patient, trends in mean costs per patient for the year prior to admission and the subsequent five years and the corresponding cost distributions across healthcare sectors. For comparison, we present corresponding estimates for the sample not admitted to short-term stays (starting from the year of the first registered COPD diagnosis in the data) and for a matched control sample constructed from the latter. We also compared the time to permanent placement in nursing home or death by presenting Kaplan–Meier curves for the three samples. We matched controls to patients admitted to short-term stays based on the latter group’s age, number of comorbidities, respiratory and non-respiratory GP contacts, in- and outpatient hospital contacts, and reception of home nursing in the year prior to admission. This was done using coarsened exact matching; to ensure that only exact multidimensional matches were included, the healthcare variables were categorized as described above. Age was stratified into groups of <65, 66–80, and >80 years, while comorbidities were categorized into 0, 1–2, and 3 or more. The health status of the matched sample should then resemble that of the short-term stay sample prior to admission. Since the ratio of controls to cases within each combination of the matching variables varied, adjustment weights were applied to the controls when calculating descriptive statistics. This adjustment was made to ensure that the ratio within each combination was identical to the ratio in the full matched sample.

Costs of services were estimated based on reimbursement fees for GP, contract specialist, physiotherapist and emergency room contacts, Diagnosis Related Group-weights for all in- and outpatient hospital care, and cost weights from an earlier pilot study for the financing of long-term care in Norway.^18^ Long-term care costs were based on hours per week for home services, and cost per day (yearly cost/365) for short-term institutional stays. All costs were standardized to 2020-levels using fees and weights for 2020 and adjusted by changes in the consumer price index (Statistics Norway) and using an exchange rate of €1.00=NOK11.75. Estimates for the cost trends are conditional on living at home at the start of each year from 1 through 5. Data were analyzed in Stata 18.0, and a 5% significance level was assumed throughout.

## Results

Basic descriptive statistics for the samples are presented in Table 2. When admitted to short-term stays, patients were on average around 10 years older than the sample with no short-term stays during follow-up, and the majority were females. Almost 70% of patients admitted to short-term stays died during the follow-up period from 2010 to 2019, compared to 19% in the group without short-term stays. The number of comorbidities was also higher among patients admitted to short-term stays. Tables 2 and S1 (in Supplementary Data) show that the matched sample resemble patients admitted to short-term stays more closely than the raw sample of patients without short-term stays even though some matching variables were categorized or not part of the matching process.

**Table 2:** Descriptive Statistics on Sociodemographic and -Economic Variables for the Total Sample, the Raw Sample with No Registered Short-Term Stay During Follow-up, the Matched Sample, and the Sample with at Least One Short-Term Stay. For the Latter Group, Statistics are Shown During the Period Before and After the First Registered Admission

Variable	Total Sample (n=24,613)	No Short-Term Stay		Short-Term Stay (n=5276)	
		Raw Sample (n=19,337)	Matched Sample (n=10,071)	Before Admission	After Admission
	Mean/% (SD)	Mean/% (SD)	Mean/% (SD)	Mean/% (SD)	Mean/% (SD)
Years of follow-up	7.3 (3.0)	7.5 (3.3)	5.7 (3.0)	5.2 (2.8)	2.1 (2.3)
Age at first COPD diagnosis in data	64.2 (11.4)	61.9 (10.9)	72.3 (10.4)	72.1 (9.9)	–
Male	47%	49%	50%	42%	–
Annual income	€38k	€42k	€33k	€29k	–
*Education*: Primary	36%	35%	40%	41%	–
Secondary	45%	45%	44%	45%	–
University/college	14%	21%	16%	14%	–
*Marital status:*					
Married/cohabitant	43%	44%	41%	36%	–
Widow/widower	13%	9%	20%	28%	–
Not married/divorced	44%	47%	38%	36%	–
Permanent disability pension	36%	38%	30%	27%	–
Permanent placement in nursing home	8%	1%	5%	–	32%
Dead during follow–up	29%	19%	49%	–	68%
Sum of comorbidities during follow–up*	3.1 (1.7)	2.9 (1.6)	4.1 (1.7)	3.7 (1.7)	4.3 (2.0)

**Note**: *Sum of the 17 comorbidity indicators listed in Table 3 (see Table S1 for definitions).

**Abbreviation**: SD, standard deviation.

**Table 3:** Multiple Regression Results of the Odds of Short-Term Stays From the Discrete Time Logit Survival Model. N=167,390 Observations From 24,613 Individuals

Outpatient Healthcare Use	Odds Ratio	Confidence Interval	p-value
GP contacts, respiratory (reference: 0)			
1-2	0.86	(0.79, 0.94)	<0.001
3-4	0.92	(0.83, 1.02)	0.11
>4	0.99	(0.91, 1.08)	0.81
			
GP contacts, non-respiratory (reference: 0–2)			
3-7	0.96	(0.86, 1.08)	0.51
8-14	0.92	(0.83, 1.03)	0.16
>14	0.78	(0.70, 0.87)	<0.001
Contract specialist, respiratory diagnoses	0.80	(0.71, 0.91)	<0.001
Contract specialist, non-respiratory diagnoses	0.81	(0.75, 0.86)	<0.001
Physiotherapy, respiratory diagnoses	0.89	(0.77, 1.02)	0.08
Physiotherapy, non-respiratory diagnoses	0.88	(0.80, 0.97)	0.01
Municipal emergency room, respiratory diagnoses	0.98	(0.91, 1.06)	0.68
Municipal emergency, non-respiratory diagnoses	1.20	(1.12, 1.29)	<0.001
Outpatient, respiratory diagnoses	1.03	(0.95, 1.11)	0.42
Outpatient, non-respiratory diagnoses	0.96	(0.88, 1.04)	0.25
Rehabilitation, respiratory diagnoses	0.87	(0.72, 1.05)	0.15
Rehabilitation, non-respiratory diagnoses	1.00	(0.88, 1.15)	0.94
**Hospital contacts**			
Hospital admissions, COPD	2.55	(2.36, 2.76)	<0.001
Hospital admissions, other	6.33	(5.81, 6.90)	<0.001
**Home care**			
Safety alarm	1.74	(1.60, 1.89)	<0.001
Any assistance at home	1.62	(1.49, 1.76)	<0.001
Assisted living	0.84	(0.70, 1.02)	0.06
Respite care	2.98	(1.95, 4.57)	<0.001
Rehabilitation out of institution	1.65	(1.49, 1.83)	<0.001
Home nursing	2.50	(2.31, 2.70)	<0.001
**Sociodemographics and -economics**			
Male	0.97	(0.91, 1.05)	0.46
Annual gross income (per €10k)	0.97	(0.96, 0.99)	<0.001
*Education* (reference: primary)			
Secondary	1.07	(0.99, 1.14)	0.06
University/college	0.95	(0.86, 1.05)	0.35
*Marital status* (reference: married/cohabitant)			
Widow/widower	1.19	(1.09, 1.30)	<0.001
Not married/divorced	1.08	(1.00, 1.17)	0.07
Permanent disability pension	1.12	(1.02, 1.24)	0.02
**Comorbidities**			
Alcoholism	2.09	(1.85, 2.37)	<0.001
Anemia	1.90	(1.72, 2.10)	<0.001
Cancer (except lung cancer)	1.37	(1.26, 1.49)	<0.001
Cardiovascular disease	1.07	(0.99, 1.16)	0.10
Dementia	2.24	(1.95, 2.56)	<0.001
Depression and anxiety	1.42	(1.29, 1.56)	<0.001
Diabetes	1.12	(1.02, 1.23)	0.02
Heart failure	1.27	(1.16, 1.38)	<0.001
Hypertension	1.11	(1.04, 1.19)	<0.001
Kidney disease or failure	1.01	(0.90, 1.14)	0.87
Lung cancer	1.68	(1.48, 1.91)	<0.001
Mental disorders	1.79	(1.53, 2.08)	<0.001
Myocardial infarction (incl. angina pectoris)	0.88	(0.79, 0.98)	0.01
Obesity	0.94	(0.73, 1.20)	0.60
Osteoporosis	1.09	(0.96, 1.23)	0.23
Stroke	1.67	(1.51, 1.85)	<0.001
Underweight	1.59	(1.47, 1.73)	<0.001

Table 3 shows the results from the regression analysis of factors associated with the likelihood of a first short-term stay. Hospital admissions and most home care services, except for assisted-living facilities, increased the odds of the outcome. The same applied to municipal emergency room visits for non-respiratory diagnoses. Outpatient care, such as yearly contacts with GP for respiratory diagnoses, being in the top 25% of GP contacts for non-respiratory diagnoses, and as contacts with contract specialists or physiotherapists suggested protective associations to the outcome. However, the magnitude of the estimated effects was modest compared with those of prior hospital admissions and home care services (hazard odds ratios between 0.78 and 0.90 compared with >1.5 for prior hospital admissions and most home care services). Among the sociodemographic and -economic variables and comorbidities, being on permanent disability pension, suffering from depression/anxiety, mental disorders or alcoholism were all associated with an increased odds of a first short-term stay. In particular, the latter three variables demonstrated substantial relative effects, comparable in magnitude to those observed for the home care service variables. The anomalous finding of a protective effect of myocardial infarction is due to the correlation between this variable, prior hospitalisations, and heart failure. The age range for the patients included in the analysis was wide (40–95 years), but restricting the analysis to observations above 67 years resulted in similar coefficients (not shown).

The corresponding univariable results are given in the Supplementary Data, Table S2. Notably, having 1–2 GP contacts for respiratory diagnoses in a year, or seeing a contract specialist for any diagnosis or a physiotherapist for other diagnoses, was associated with reduced odds of a first short-term stay even without adjusting for comorbidities and other healthcare use.

Table 4 presents descriptive statistics on healthcare use and costs for the raw and matched samples of patients with no registered short-term stay during follow-up and the sample with at least one short-term stay. The average use of most healthcare services per year per patient before the first short-term stay was similar to that in the raw sample without short-term stays. The most notable differences were in hospital admissions for non-respiratory diagnoses and the use of home care services. Comparing the time periods before and after the first short-term stay, there was a notable increase in the use of services related to non-respiratory diagnoses and long-term care. On average, home care and short-term institutional costs increased by approximately €10,000 and €5000 per year per patient, respectively, after the first short-term stay. Compared to the raw sample with no registered short-term stay, the matched sample was relatively similar to those admitted to short-term stays also in terms of the number of hospital admissions, the prevalence of home services and costs calculated across all follow-up years prior to admission. However, the matched sample still differed markedly compared to the period following the short-term stay.

**Table 4:** Descriptive Statistics on Healthcare Use and Costs for the Sample with No Registered Short-Term Stay During Follow-up, the Matched Sample, and the Sample with at Least One Short-Term Stay. For the Latter Group, Statistics are Shown During the Period Before and After the First Registered Admission

	No Short-Term Stay		Short-Term Stay (n=5276)	
	Raw Sample (n=19,337)	Matched Sample (n=10,071)	Before Admission	After Admission
**Outpatient healthcare use**	Mean per year per patient (SD)			
GP contacts, respiratory diagnoses	3.1 (3.4)	3.2 (5.7)	3.0 (3.9)	3.4 (4.8)
GP contacts, non-respiratory diagnoses	9.2 (8.1)	12.9 (12.6)	11.9 (10.0)	14.9 (11.8)
Contract specialist, respiratory diagnoses	0.3 (1.0)	0.3 (1.1)	0.2 (0.6)	0.1 (0.3)
Contract specialist, non-respiratory diagnoses	1.2 (3.1)	1.5 (3.0)	1.5 (2.7)	1.0 (1.6)
Physiotherapy, respiratory diagnoses	1.6 (7.0)	1.7 (6.4)	1.5 (6.7)	1.0 (4.3)
Physiotherapy, non-respiratory diagnoses	3.1 (9.4)	3.9 (11.6)	3.2 (8.8)	3.8 (8.2)
Municipal emergency room, respiratory diagnoses	0.2 (0.6)	0.4 (0.9)	0.3 (0.8)	0.5 (1.0)
Municipal emergency room, non-respiratory diagnoses	0.5 (1.6)	1.0 (2.6)	0.7 (1.8)	1.4 (3.1)
Outpatient, respiratory diagnoses	0.4 (1.4)	0.4 (1.0)	0.5 (1.9)	0.5 (1.3)
Outpatient, non-respiratory diagnoses	2.5 (4.1)	3.7 (5.4)	3.2 (4.2)	3.8 (6.9)
Rehabilitation, respiratory diagnoses	0.1 (0.4)	0.1 (0.4)	0.1 (0.5)	0.1 (0.4)
Rehabilitation, non-respiratory diagnoses	0.2 (0.8)	0.2 (1.1)	0.4 (0.7)	2.2 (2.9)
**Inpatient hospital admissions**	Mean per year per patient (SD)			
Hospital admissions, COPD	0.1 (0.4)	0.3 (0.8)	0.4 (0.8)	0.5 (1.3)
Hospital admissions, other diagnoses	0.5 (1.5)	1.0 (2.2)	1.1 (3.0)	1.5 (4.1)
**Home care**	Cumulative prevalence during follow-up			
Safety alarm	11%	35%	48%	66%
Any assistance at home	13%	37%	49%	65%
Assisted living	1%	5%	4%	8%
Respite care	<1%	1%	1%	4%
Rehabilitation out of institution	7%	23%	22%	32%
Home nursing	20%	62%	62%	84%
**Costs (per €1000)**	Mean per year per patient (SD)			
Total costs	4.8 (12.8)	11.4 (20.4)	12.2 (22.7)	30.5 (33.6)
Outpatient costs	1.4 (5.2)	2.1 (6.0)	2.2 (9.8)	1.8 (2.9)
Inpatient costs	2.4 (7.8)	5.7 (12.0)	5.6 (13.2)	9.6 (16.8)
Home care costs	0.9 (7.4)	3.6 (12.0)	4.3 (13.9)	13.8 (25.3)
Short-term institutional costs	–	–	–	5.3 (12.9)

**Abbreviation**: SD, standard deviation.

**Figure 1:**
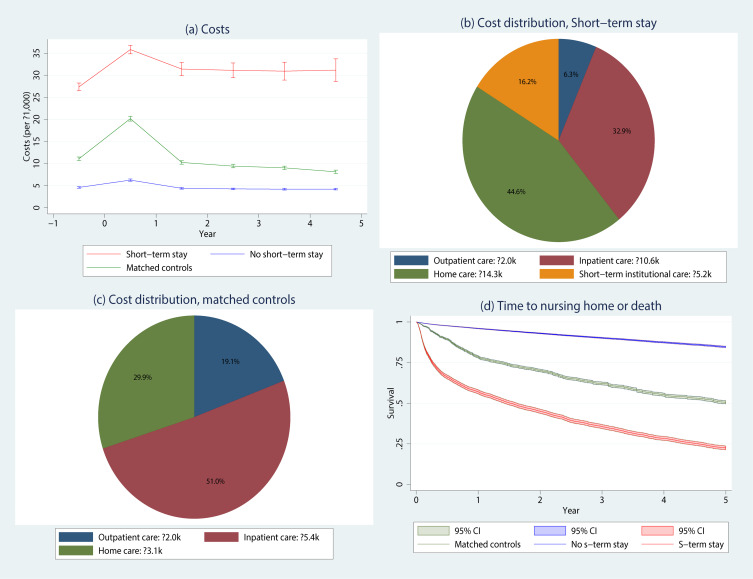
Total mean costs per year (**a**), the corresponding cost distribution for the sample with at least one short-term stay during follow-up (**b**) and the matched sample (**c**), and Kaplan–Meier curves for the time to nursing home or death (**d**).

**Notes**: Graphs (**a** and **d**): Year 0 corresponds to the time of first observed COPD diagnosis (raw and matched samples with no short-term stays) and the time of first short-term stay, respectively. 95% pointwise confidence intervals (CI) are included. Diagrams (**b** and **c**): Outpatient care=GP, emergency room, physiotherapist, contract specialist, outpatient and rehabilitation hospital contacts.

Figure 1a illustrates cost trends as means per patient over six years of follow-up, along with the corresponding cost distribution across healthcare sectors in diagrams (b) and (c). The cost difference over time was substantial when compared to both the raw sample not admitted to short-term stays and the matched sample (Figure 1a). The matched sample exhibited high costs during the first year (the year of matching), but these costs decreased rapidly in the second and subsequent years. This trend was quite different from that observed in the sample admitted to short-term stays, where costs remained high in all subsequent years. The initial cost discrepancy between the matched and short-term stay samples is attributed to a substantial increase in costs in the year prior to the first short-term stay. Comparing cost distributions across the same years, we see that the costs incurred by the sample admitted to short-term stays is dominated by home care services and to a lesser extent by short-term institutional care (Figure 1b). In contrast, for the matched sample, inpatient hospital costs account for the largest share (Figure 1c). Across all six years, the estimated costs for those admitted to a first short-term stay were around €22,000 higher per year per patient than for the matched controls (from Figure 1b and c). Figure 1d also depicts the time to nursing home admission or death for the three samples. The difference in the proportion of individuals living at home or surviving after five years is substantial, approximately 25%, when comparing the matched sample to patients admitted to short-term stays.

## Discussion

### Main Findings

The indicators for depression/anxiety, mental illnesses, and alcoholism were all associated with increased odds of a short-term stay. These factors are related to lower socio-economic status and social vulnerability. Most hospital and municipal services were associated with an increased likelihood of a short-term stay, probably due to the poorer health typically experienced by those who require these services. As noted in the Introduction, short-term stays are frequently used in the transition from hospital to home-based care. Use of outpatient services, including GP, specialist, and physiotherapy contacts, were to some extent associated with reduced odds. These observations suggest that patients with better prognoses are more often managed out of long-term care, in keeping with the principles of prioritization in Norway. The results further showed markedly higher costs one year prior to and five years after the first short-term stay, as well as significantly shorter time to permanent placement in nursing home or death, compared with matched control patients. These findings suggest that the service is indeed provided for the patients with the highest need for healthcare. The increase in costs following a short-term stay were due to increases both in costs of in-hospital care, home-based care and repeated short-term institutional stays. Compared with the matched sample, costs for patients admitted to short-term stays remained high for several years, whereas the matched patients incurred the highest costs only in the year used for matching.

### Socio-Economic Status and Social Vulnerability

Several factors related to social vulnerability and lower socioeconomic status are more prevalent among COPD patients than in the general population.^1^,^19^,^20^ Both anxiety and depression may be associated with symptom experience and disease severity,^21^ putting these patients at a higher risk of exacerbations and mortality.^22^,^23^ One might fear that these patients may be less likely to seek long-term care from the municipality, making it more difficult for municipal health providers to identify and assist them. Consequently, they may be less likely to receive services that meet their needs. An important finding is thus that patients on permanent disability pension, or those suffering from depression/anxiety, mental disorders, or alcoholism, have a higher likelihood of short-term stays even after adjusting for other healthcare use and comorbidities. Conversely, it could be that these patients do not receive adequate care at an earlier stage, which might explain the increased odds of short-term stays observed for these factors in this study. However, a previous analysis showed that the same factors were also associated with an increased likelihood of receiving any type of service from the municipality.^15^ Additionally, further analyses on the short-term stay sample indicated that neither depression/anxiety, mental disorders, alcoholism nor permanent disability pension were significant predictors for the time to permanent placement in nursing home or death. Hence, it appears that socially vulnerable patients do not receive municipal services or are admitted to short-term stays later than other COPD patients.

### Effects of Outpatient Service Use

As noted in the Introduction, there is a lack of studies on the association between outpatient services and becoming a recipient of long-term care, both for COPD patients and in general. The reduced odds for short-term stays observed for yearly contacts with a GP for respiratory diagnoses, a high number of contacts for other diagnoses, as well as contacts with contract specialists and physiotherapy, could suggest preventive effects of outpatient care for this patient group. However, since the data are observational, and the associations are not necessarily causal, such conclusions should be made with caution. We are not aware of any similar findings for the COPD population, but an association between outpatient care and lower hospital costs has been found in an earlier study in a general sample of patients near the end of life.^24^ The results also showed that most home care services were associated with increased odds of short-term stays. It may be that when the patient is in sufficient health, healthcare is more likely to be provided by the GP, a contract specialist, and a physiotherapist. Consequently, these factors are associated with reduced odds of short-term stays. Since home care services were relatively common even before the first short-term stay, it appears that the municipality is involved in the care process also prior to a patient’s admission to a short-term stay institution. This makes it difficult to conclude how frail patients could be identified earlier in the care process based on the analysis performed here.

### Healthcare Costs

As far as we know, this is the first study describing trends in cost composition for COPD patients before and after the reception of long-term care in general. Previous research has focused on the macro-costs^3–5^ or the incremental costs for a COPD population compared to non-COPD patients,^10^,^11^,^25^,^26^ and not the changes in the use of services and costs by type of provider over time. A large variation in healthcare costs in the COPD population has been found also in the U.S.^27^ Although the total costs were relatively similar in the short-term stay sample and matched sample when comparing the average across all follow-up years prior to the short-term stay (Table 4), it is apparent that the trends were very different. The costs were significantly higher already in the year prior to the first short-term stay and continued at a high level in the following years. The matched sample had the highest costs in the year of first observed COPD diagnosis, possibly reflecting a worsening of the disease with high inpatient and home care costs during that particular year, but significantly lower costs in the following years. The increase in home care costs after the first short-term stay could be caused by additional care needs identified during the stay or a worsening of COPD or comorbid diagnoses. A similar argument could be made for the higher inpatient hospital costs observed after the first short-term stay. A previous study from the US has found that discharge to long-term care was associated with high-cost hospital care.^28^

In conclusion, the analyses of costs and time to nursing home placement or death suggest that when short-term institutional stays are provided, the patient’s healthcare needs are already substantial. Therefore, if the goal is to improve the quality of care in order to delay or reduce later use of healthcare resources and prolong survival for COPD patients, preventive care should likely be initiated at an earlier stage in the care process. However, it is important to recognise that even if patient costs are reduced or delayed in the short term, they may still increase at a later age, potentially reaching levels similar to those observed in our analysis.

### Strengths and Limitations

The main strength is the extensive content in the data, and the fact that they are linked at the individual level. This allows for analyses of health service use over time across providers at different levels, rarely possible due to the lack of such registry data in most countries. Variables on health service use may both be used as covariates in the association to outcomes, to describe trends in healthcare use and costs over time, and to some extent for capturing health status in regression risk adjustment or matching. However, medication data were not available. Previous estimates indicate that they make up around a third of the total direct healthcare costs.^3^ The main limitation is that it is difficult to make any causal conclusions from the analyses. In the estimated associations to short-term stays, further limitations were the lack of information on smoking status and clinical measures of the COPD severity. Smoking is one of the most important risk factors for COPD,^1^ and as this is a general sample of COPD patients, there is also great heterogeneity in the COPD severity. However, both of these factors are generally not registered in administrative data, and as such are limitations not specific to the analyses presented here. In the regression analyses, we used a discrete time model with independent variables updated per year of follow-up, rather than a continuous time survival analysis. This approach was chosen due to the large number of time-dependent variables present in the data. Another possible limitation is that only two cities are included. This may reduce generalizability, as the capacity and breadth in available services varies across Norway. On the other hand, the patients’ rights to long-term care services are stated in laws and regulations at the national level, to be followed by all municipalities.

## Conclusion

This study’s first aim was to identify factors associated with higher or lower likelihood of admission to a first short-term institutional stay. Our results showed that factors related to social vulnerability were associated with a higher odds of admission to a first short-term stay. The same applied to prior hospitalization and use of home care services. This suggests that the resource allocation follows the need in the two cities, at least when focusing on the population level and not the individual patient. The use of outpatient services, such as GP, specialists or physiotherapists, was to some extent associated with lower odds of admission to a first short-term stay. Although these findings are intriguing, further analyses are needed in order to establish whether these effects are causal. The second aim was to compare the healthcare use, costs, and time living at home for patients admitted to a first short-term stay versus patients not admitted. Our results showed that costs were higher for patients admitted to short-term stays compared to similar patients who were not admitted during follow-up, both in the year before the first admission and in the subsequent years. This suggests that by the time a first short-term stay is required, the healthcare needs of the patient are already significant due to factors not captured in this analysis.

## Data Availability

The datasets used in the current study are based on national registries and are not publicly available. Access to pseudonymised data from the national registries are only granted through application to the Norwegian Centre for Research Data and Regional Committees for Medical and Health Research Ethics.

## References

[1] Global initiative for chronic obstructive lung disease (GOLD): global strategy for the diagnosis, management and prevention of chronic obstructive pulmonary disease, 2023. Available from: https://goldcopd.org/2023-gold-report-2/. Accessed February 4, 2025.

[2] Chen S, Kuhn M, Prettner K, et al. The global economic burden of chronic obstructive pulmonary disease for 204 countries and territories in 2020-50: a health-augmented macroeconomic modelling study. *Lancet Glob Health*. 2023;11(8):e1183–e1193. doi:10.1016/S2214-109X(23)00217-6 37474226 PMC10369014

[3] European Respiratory Society. The economic burden of lung disease. In: *European Lung White Book: Respiratory Health and Disease in Europe*. Brussels: World Health Organization; 2013.

[4] Duan KI, Birger M, Au DH, Spece LJ, Feemster LC, Dieleman JL. Health care spending on respiratory diseases in the United States, 1996-2016. *Am J Respir Crit Care Med*. 2023;207(2):183–192. doi:10.1164/rccm.202202-0294OC 35997678 PMC9893322

[5] Kinge JM, Dieleman JL, Karlstad Ø, et al. Disease-specific health spending by age, sex, and type of care in Norway: a national health registry study. *BMC Med*. 2023;21(1):201. doi:10.1186/s12916-023-02896-6 37277874 PMC10243068

[6] Momtazmanesh S, Moghaddam SS, Ghamari S-H, GBD 2019 Chronic Respiratory Diseases Collaborators. Global burden of chronic respiratory diseases and risk factors, 1990-2019: an update from the global burden of disease study 2019. *EClinicalMedicine*. 2023;59:101936. doi:10.1016/j.eclinm.2023.101936 37229504 PMC7614570

[7] Adeloye D, Song P, Zhu Y, Campbell H, Sheikh A, Rudan I, NIHR RESPIRE Global Respiratory Health Unit. Global, regional, and national prevalence of, and risk factors for, chronic obstructive pulmonary disease (COPD) in 2019: a systematic review and modelling analysis. *Lancet Respir Med*. 2022;10(5):447–458. doi:10.1016/S2213-2600(21)00511-7 35279265 PMC9050565

[8] Faes K, De Frène V, Cohen J, Annemans L. Resource use and health care costs of COPD patients at the end of life: a systematic review. *J Pain Symptom Manage*. 2016;52(4):588–599. doi:10.1016/j.jpainsymman.2016.04.007 27401511

[9] Simoni-Wastila L, Blanchette CM, Qian J, et al. Burden of chronic obstructive pulmonary disease in medicare beneficiaries residing in long-term care facilities. *Am J Geriatr Pharmacother*. 2009;7(5):262–270. doi:10.1016/j.amjopharm.2009.11.003 19948302

[10] D’Souza AO, Shah M, Dhamane AD, Dalal AA. Clinical and economic burden of COPD in a medicaid population. *COPD*. 2014;11(2):212–220. doi:10.3109/15412555.2013.836168 24111752

[11] Miller JD, Foster T, Boulanger L, et al. Direct costs of COPD in the U.S.: an analysis of Medical Expenditure Panel Survey (MEPS) data. *COPD*. 2005;2(3):311–318. doi:10.1080/15412550500218221 17146996

[12] Kim J, Lee TJ, Kim S, Lee E. The economic burden of chronic obstructive pulmonary disease from 2004 to 2013. *J Med Econ*. 2016;19(2):103–110. doi:10.3111/13696998.2015.110011 26414920

[13] Schmidt-Mende K, Arvinge C, Cioffi G, Gustafsson LL, Modig K, Meyer AC. Profiling chronic diseases and hospitalizations in older home care recipients: a nationwide cohort study in Sweden. *BMC Geriatr*. 2024;24(1):312. doi:10.1186/s12877-024-04796-7 38570768 PMC10993481

[14] Norwegian Directorate of Health. Helse-, omsorgs- og rehabiliteringsstatistikk - Eldres helse og bruk av kommunale helse- og omsorgstjenester [Health, care, and rehabilitation statistics - Elderly health and use of municipal health and care services]. 2016. Report no. 2/2016.

[15] Moger TA, Holte JH, Amundsen O, et al. Describing the in- and outpatient health care use of patients with COPD before and after reception of home care services: a registry study from Norway. *Scand J Prim Health Care*. 2024;16:1–11. doi:10.1080/02813432.2024.2404056 PMC11834786 39282877

[16] Moger TA, Amundsen O, Tjerbo T, Hellesø R, Holte JH, Vøllestad NK. Innovations in use of registry data (INOREG) - Design of a registry-based study analyzing care pathways and outcomes for chronic patients. *NJHE*. 2023;6:129–146.

[17] Norwegian Directorate of Health. Nasjonal faglig retningslinje for diagnostisering og behandling av kronisk obstruktiv lungesykdom (kols) [National guideline for the diagnosis and treatment of chronic obstructive pulmonary disease (COPD)]. 2012. IS–2029. Updated in 2022.

[18] Norwegian Directorate of Health. Forsøk med statlig finansiering av omsorgstjenester [Trial with state financing of care services]. 2018. Report no. 12/2018.

[19] Schneider C, Jick SS, Bothner U, Meier CR. COPD and the risk of depression. *Chest*. 2010;137(2):341–347. doi:10.1378/chest.09-0614 19801582

[20] Hynninen KM, Breitve MH, Wiborg AB, Pallesen S, Nordhus IH. Psychological characteristics of patients with chronic obstructive pulmonary disease: a review. *J Psychosom Res*. 2005;59(6):429–443. doi:10.1016/j.jpsychores.2005.04.007 16310027

[21] Spitzer C, Gläser S, Grabe HJ, et al. Mental health problems, obstructive lung disease and lung function: findings from the general population. *J Psychosom Res*. 2011;71(3):174–179. doi:10.1016/j.jpsychores.2011.03.005 21843753

[22] Rahi MS, Thilagar B, Balaji S, et al. The impact of anxiety and depression in chronic obstructive pulmonary disease. *Adv Respir Med*. 2023;91(2):123–134. doi:10.3390/arm91020011 36960961 PMC10037643

[23] Abrams TE, Vaughan-Sarrazin M, Van der Weg MW. Acute exacerbations of chronic obstructive pulmonary disease and the effect of existing psychiatric comorbidity on subsequent mortality. *Psychosomatics*. 2011;52(5):441–449. doi:10.1016/j.psym.2011.03.005 21907063

[24] Kronman AC, Ash AS, Freund KM, Hanchate A, Emanuel EJ. Can primary care visits reduce hospital utilization among medicare beneficiaries at the end of life? *J Gen Intern Med*. 2008;23(9):1330–1335. doi:10.1007/s11606-008-0638-5 18506545 PMC2518010

[25] Bilde L, Rud Svenning A, Dollerup J, Baekke Borgeskov H, Lange P. The cost of treating patients with COPD in Denmark--a population study of COPD patients compared with non-COPD controls. *Respir Med*. 2007;101(3):539–546. doi:10.1016/j.rmed.2006.06.020 16889949

[26] Mannino DM, Roberts MH, Mapel DW, et al. National and local direct medical cost burden of COPD in the United States from 2016 to 2019 and projections through 2029. *Chest*. 2024;165(5):1093–1106. doi:10.1016/j.chest.2023.11.040 38042365

[27] Iyer AS, Goodrich CA, Dransfield MT, et al. End-of-life spending and healthcare utilization among older adults with chronic obstructive pulmonary disease. *Am J Med*. 2020;133(7):817–824.e1. doi:10.1016/j.amjmed.2019.11.024 31883772 PMC7319886

[28] Mulpuru S, McKay J, Ronksley PE, Thavorn K, Kobewka DM, Forster AJ. Factors contributing to high-cost hospital care for patients with COPD. *Int J Chron Obstruct Pulmon Dis*. 2017;12:989–995. doi:10.2147/COPD.S126607 28392683 PMC5373828

